# Protein phosphatase PP2C19 controls hypocotyl phototropism through the phosphorylation modification of NONPHOTOTROPIC HYPOCOTYL3 in *Arabidopsis*

**DOI:** 10.1093/pcp/pcae141

**Published:** 2024-11-28

**Authors:** Tatsuya Sakai, Ken Haga, Taro Kimura, Keita Kawaura

**Affiliations:** Graduate School of Science and Technology, Niigata University, Niigata 950-2181, Japan; Department of Applied Chemistry, Faculty of Fundamental Engineering, Nippon Institute of Technology, Saitama 345-8501, Japan; Graduate School of Science and Technology, Niigata University, Niigata 950-2181, Japan; Graduate School of Science and Technology, Niigata University, Niigata 950-2181, Japan

**Keywords:** *Arabidopsis*, phototropism, PP2C, NPH3, phosphorylation

## Abstract

Plants exhibit shoot growth in the direction of the light source to facilitate photosynthesis, known as positive phototropism. In *Arabidopsis* hypocotyl phototropism, it is thought that a gradient of the signal intensity of the blue light (BL) photoreceptor phototropin1 (phot1) between the light-irradiated and shaded sides leads to the differential growth of hypocotyls. The intensity of phot1 signal is regulated not only by the protein kinase activity of phot1 but also by the phosphorylation status of the NONPHOTOTROPIC HYPOCOTYL3 (NPH3) protein, which has a dark form and a BL form of the phosphorylation modification. Previous studies have shown that phot1 drives the forward reaction from the dark form to the BL form of NPH3. However, the molecular mechanism underlying the reverse reaction remains unknown. Here, we show that protein phosphatase PP2C19 controls the reverse reaction that converts the BL form of NPH3 to the dark form of NPH3. The PP2C19 protein possesses the protein phosphatase type 2C (PP2C) domain, two cyclic nucleoside monophosphate (cNMP)-binding domains, and the protein kinase domain. Similar to phot1 and NPH3, PP2C19 localizes to the plasma membrane, and its PP2C domain is necessary and sufficient for PP2C19 function in hypocotyl phototropism. The *pp2c19* mutants show abnormalities in second positive hypocotyl phototropism with a delay in the reverse reaction of NPH3 phosphorylation modification. The present study suggests that continuous BL irradiation induces an equilibrium state of the reversible reaction of NPH3 phosphorylation, which acts as a phot1 signaling gradient with phot1 kinase activity to induce the second positive phototropism.

## Introduction

Plants exhibit positive phototropism, a phenomenon in which the aboveground parts of a plant grow in the direction of the light to facilitate photosynthesis (Poff et al. 1994, Iino 2001, Briggs 2014, Haga and Sakai 2023). The phototropic responses of etiolated dicot hypocotyls and etiolated monocot coleoptiles in response to different durations of unilateral blue light (BL) irradiation occur in two distinct patterns (Poff et al. 1994, Iino 2001, Haga and Sakai 2023). One is pulse-induced, first positive phototropism. In *Arabidopsis thaliana* (*Arabidopsis*), this is induced by unilateral BL irradiation with a short pulse of ≤3 min followed by dark incubation (Haga and Sakai 2012, Haga et al. 2014). The pulse-induced phototropic signal is memorized for a certain period to induce hypocotyl curvature in the subsequent dark incubation. The fluence-response curve of first positive phototropism shows a typical bell shape. With increasing light fluence, the magnitude of the response decreases, and a refractory state is reached. The other is second positive phototropism, which is typically induced with continuous BL irradiation. In particular, when we analyze the recovery of second positive phototropism from the refractory state in the fluence-response curve, we observe the second positive phototropism using prolonged BL stimulation (e.g. 9–27 min) and subsequent dark incubation (Iino 2001, Haga and Sakai 2012). We call this time-dependent phototropism, in distinction to continuous light-induced phototropism (Iino 2001, Haga and Sakai 2012, Haga et al. 2014, 2015).

The phototropic responses are induced by the activation of BL photoreceptors—phototropins (phots; Christie 2007). Phots localize on the inner surface of the plasma membrane and exhibit Ser/Thr kinase activity under BL conditions. *Arabidopsis* has two phots, phot1 and phot2, of which phot1 functions as the major photoreceptor for phototropic responses (Sakai et al. 2001, Sakai and Haga 2012, Kimura et al. 2022). Previous studies have identified several signaling molecules that interact with phot1. One such molecule is NONPHOTOTROPIC HYPOCOTYL3 (NPH3; Motchoulski and Liscum 1999), which belongs to the NPH3/ROOT PHOTOTROPISM2 (RPT2)-like family with RPT2 (Christie et al. 2018). It binds to phot1 and modulates phot1 signaling in response to phot1 activity and the corresponding phosphorylation status of NPH3 (Motchoulski and Liscum 1999, Haga et al. 2015, Sullivan et al. 2019, 2021, Kimura et al. 2021). The Ser 744 residue (S744) of NPH3 is phosphorylated by BL-activated phot1, and its phosphorylation is significant for second positive phototropism (Sullivan et al. 2021). Several Ser residues in intrinsically disordered regions (IDRs) of NPH3—including S213, S223, and S237—are phosphorylated in the dark and are dephosphorylated by BL irradiation in a phot1-dependent manner (Pedmale and Liscum 2007, Tsuchida-Mayama et al. 2008, Kimura et al. 2021). Dephosphorylation in IDRs is crucial for the recovery of second positive phototropism from the refractory state under high-intensity BL conditions (Haga et al. 2015, Kimura et al. 2021). Thus, phot1 drives the conversion of the NPH3 protein from the S744-unphosphorylated, IDR-phosphorylated form (dark form) to the S744-phosphorylated, IDR-dephosphorylated form (BL form) by BL irradiation. Stopping BL irradiation leads to the reverse reaction that facilitates the conversion of the BL form of NPH3 protein to the dark form (Pedmale and Liscum 2007, Sullivan et al. 2021); however, the molecular mechanisms underlying the reverse reaction remain largely unknown.

RPT2 also has a protein–protein interaction with phot1. Light-inducible RPT2 proteins bind to phot1 and suppress its autophosphorylation activity to maintain phot1 in a moderate activated state under any light intensity (Sakai et al. 2000, Inada et al. 2004, Haga et al. 2015, Kimura et al. 2020). Wild-type seedlings show a saturation of the forward reaction of NPH3 phosphorylation during the refractory state of the fluence-response curve (Haga et al. 2015). RPT2 suppresses phot1 activity and leads to an equilibrium between the dark and BL forms of NPH3 proteins, resulting in the recovery of second positive phototropism under high-intensity BL (Haga et al. 2015, Kimura et al. 2020). Even under low-intensity BL irradiation at 0.0017 μmol m^−2^ s^−1^, a pre-existing expression of RPT2 induced by red light (RL) pretreatment is essential for time-dependent phototropism (Haga et al. 2015). These results suggest that a phot1 hyperactivation due to the absence of RPT2 enhances the forward reaction of NPH3 phosphorylation, resulting in an equilibrium shift toward the BL form of NPH3 protein and a defective second positive phototropic response.

Auxin efflux carriers such as PIN-FORMED3 (PIN3) and PIN7 are required for first positive phototropism (Haga and Sakai 2012). AGC (for cAMP-dependent, cGMP-dependent, and protein kinase C) kinases—including D6 PROTEIN KINASE (D6PK) and AGC1-12—and PROTEIN PHOSPHATASE 6 (PP6) modify the phosphorylation status of PINs (Dai et al. 2012, Zourelidou et al. 2014, Haga et al. 2018), and their mutants, the *d6pk* and *agc1-12* single mutants and the *fypp1 fypp3* double mutant (*FYPP1*—that is, *PHYTOCHROME-ASSOCIATED SERINE/THREONINE PROTEIN PHOSPHATASE 1—*and *FYPP3* encode the catalytic subunit of PP6), show a hypocotyl phototropism phenotype similar to that of the *pin3 pin7* double mutant (Haga et al. 2018, Haga and Sakai 2018). Previous studies suggested that transient phot1 signaling in response to pulse light irradiation leads to the formation of a gradient of some molecular labeled PIN3 and PIN7 proteins in the light-irradiated and shaded sides of hypocotyls. This gradient serves as a memory of the direction of the light source and leads to the formation of an asymmetric auxin gradient in the subsequent dark incubation. In this context, the phosphorylation modification of PINs is a potential molecular label associated with the memory of the direction of a light source. Conversely, it has been reported that continuous BL irradiation induces a normal second positive phototropic response in *pin3 pin7* double mutants (Haga and Sakai 2012). This result suggests that if the phot1 signaling gradient is maintained under continuous BL irradiation, the phototropic response can be demonstrated even in the absence of PIN3 and PIN7.

In the present study, we explored the molecular mechanisms of PIN3/PIN7-independent phototropic responses by investigating the *pin3 pin7* enhancer mutants, which show a defect in second positive phototropism under the continuous BL condition. We found that a loss-of-function mutation in the *PP2C19* gene—which encodes a protein containing the protein phosphatase type 2C (PP2C) domain, two cyclic nucleoside monophosphate (cNMP)-binding domains, and the protein kinase domain—causes a defect in second positive phototropism in the *pin3 pin7* mutant. Our results suggest that PP2C19 promotes the reverse reaction of NPH3 phosphorylation and that a defect of *PP2C19* results in an abnormal second positive phototropic response in *Arabidopsis* hypocotyls.

## Results

### Screening of *pin3 pin7* enhancer mutants based on continuous light-induced phototropism

**Figure 1.**
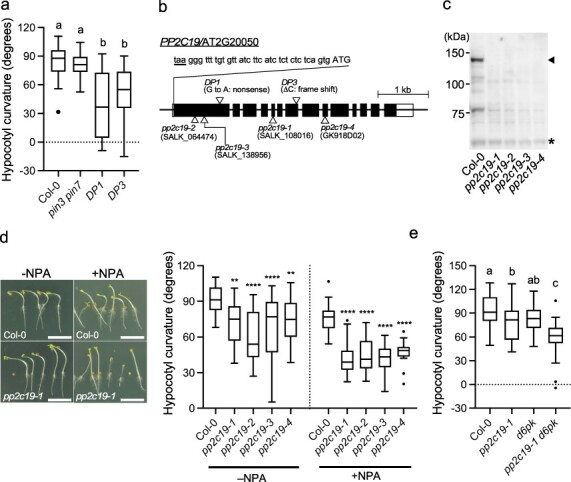
Isolation of *DP1* and *DP3* mutants and identification of the responsible gene. (a) Phototropic responses of etiolated hypocotyls of wild-type (Col-0) seedlings and *pin3 pin7*, *DP1*, and *DP3* mutants. Two-day-old etiolated seedlings grown along the surface of vertically oriented agar medium were irradiated with unilateral BL at 0.17 µmol m^–2^ s^–1^ for 6 h. Hypocotyl curvatures of 34–47 seedlings were measured. The box plots show the median (center line), quartiles (box limits), minimum–maximum values (whiskers), and outliers [dots: values located outside 1.5 times IQR (IQR: the interquartile range between the first and the third quartiles)]. Different letters (a and b) indicate statistically significant differences, and the same letters mark statistically nonsignificant differences (Tukey–Kramer multiple comparisons test, *P* < .05). (b) Genome structure of *PP2C19*. Black rectangles indicate protein-coding regions and white rectangles indicate noncoding regions of *PP2C19* exons. The start codon (ATG) was identified downstream of a nonsense codon (taa) in the 5ʹ region of the longest open reading frame. Triangles indicate the sites of the point mutations in *DP1* and *DP3* and the T-DNA insertion sites in *pp2c19-1*, *pp2c19-2*, *pp2c19-3*, and *pp2c19-4*. (c) Immunoblotting of the PP2C19 protein. Total protein extracts of 2-day-old etiolated seedlings of Col-0, *pp2c19-1*, *pp2c19-2*, *pp2c19-3*, and *pp2c19-4* were analyzed: 20 μg of each protein preparation was separated by 6% SDS-PAGE and immunoblotted using anti-PP2C19 antibodies. The arrowhead indicates the PP2C19 protein, and the asterisk indicates a nonspecific band. (d) Effect of NPA on the hypocotyl phototropism of *pp2c19* mutants. Two-day-old etiolated seedlings grown along the surface of vertically oriented agar medium with or without 1 µM NPA (+NPA and −NPA, respectively) were irradiated with unilateral BL at 0.17 µmol m^–2^ s^–1^ for 6 h. Hypocotyl curvatures of 21–39 seedlings were measured. The box plots’ components are described in (a). Asterisks indicate significant differences compared with the Col-0 (**P < .01; ****P < .0001) as determined by one-way analysis of variance followed by Dunnett’s multiple comparison test. Left panels show typical phototropic responses of Col-0 and *pp2c19-1* seedlings on the agar medium with or without NPA. Scale bar = 5 mm. (e) Phototropic responses of etiolated hypocotyls of wild-type (Col-0) seedlings and *pp2c19-1*, *d6pk*, and *pp2c19-1 d6pk* mutants. Two-day-old etiolated seedlings grown along the surface of vertically oriented agar medium were irradiated with unilateral BL at 0.17 µmol m^–2^ s^–1^ for 6 h. Hypocotyl curvatures of 41–57 seedlings were measured. The box plots’ components are described in (a). Different letters (a–c) indicate statistically significant differences, and the same letters indicate statistically nonsignificant differences (Tukey–Kramer multiple comparisons test, *P* < .05).

Our previous study showed that the *pin3 pin7* double mutant exhibits normal phototropic responses under continuous BL conditions (Haga and Sakai 2012). To identify a PIN3-/PIN7-independent pathway for the induction of phototropic responses, we isolated mutants showing a defect in continuous light-induced hypocotyl phototropism from ∼34 000 *pin3-5 pin7* (Salk_048791) M_2_ seeds treated with ethyl methanesulfonate (EMS). Mutants showing a defect in the expression of PHOT1, NPH3, or RPT2 proteins in immunoblot analyses were excluded from screening, as these mutants were predicted to be alleles of *phot1, nph3*, or *rpt2*. Two-day-old etiolated seedlings of selected mutants, *DP1* and *DP3*, showed a decrease in hypocotyl curvature in second positive phototropism; however, seedlings of their parental line, *pin3 pin7*, showed a similar curvature to that of Col-0 wild-type seedlings on the surface of vertically oriented agar medium (Fig. 1a). *DP3* was crossed with the *pin3* (ET5573) *pin7-3* double mutant (Landsberg *erecta* background), and the F_2_ segregating population was used for positional cloning of the responsible gene. The responsible gene was mapped to a region of ∼0.9 Mb between the simple sequence length polymorphism (SSLP) markers F3P11 and F7D8 on chromosome 2 (Supplementary Fig. S1). Whole-genome sequencing was performed for *DP1* and *DP3*, and it was found that the *PP2C19* gene, located between SSLP markers F3P11 and F7D8, has loss-of-function mutations in *DP1* and *DP3* genomes.

The *PP2C19* gene, annotated as the AGI code AT2G20050, consists of 14 exons [Fig. 1b; The Arabidopsis Information Resource (TAIR): https://www.arabidopsis.org] and encodes a protein of 1094 amino acid residues with a predicted molecular mass of 121 kDa. The *DP1* and *DP3* mutants had a nonsense mutation at residue 296 (nucleotide sequence changed from TGG to TGA) and a frame-shift mutation at residue 637 (single-nucleotide deletion of C from TGC), respectively (Fig. 1b). Four T-DNA insertion lines—*pp2c19-1* (SALK_108016), *pp2c19-2* (SALK_064474), *pp2c19-3* (SALK_138956), and *pp2c19-4* (GK918D02)—were obtained from the Arabidopsis Biological Resource Center (ABRC; Fig. 1b). Their homozygous mutants showed no expression of PP2C19 proteins in immunoblot analysis performed using anti-PP2C19 antibody (Fig. 1c). Analysis of second positive phototropism showed that all these mutants showed a slight decrease in hypocotyl curvature (Fig. 1d). Instead of mutations in *pin3* and *pin7*, we examined the effect of the auxin transport inhibitor *N*-1-naphthylphthalamic acid (NPA) or the *d6pk* mutation on the phototropic responses in the *pp2c19* mutants. The inhibitory effect of 1 μM NPA on the phototropic responses was greater in the *pp2c19* mutants than in the wild-type seedlings (Fig. 1d). The *d6pk* mutant showed normal second positive phototropism, whereas the *d6pk* mutation suppressed the phototropic response in the *pp2c19* mutants (Fig. 1e). Taken together, these findings suggest that the gene responsible for the *DP1* and *DP3* phenotypes is the *PP2C19* gene.

### Functional analysis of PP2C19 protein domains

**Figure 2.**
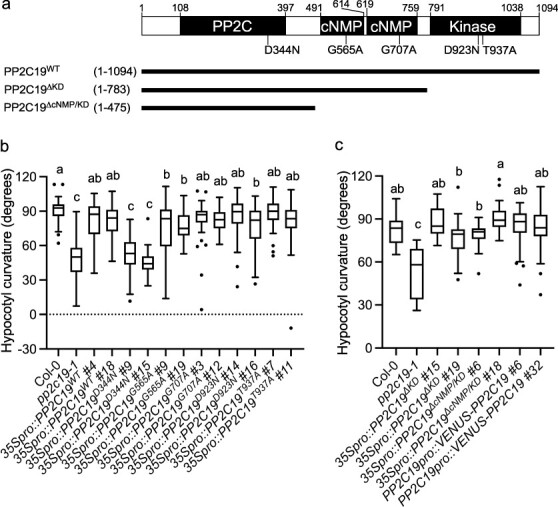
Functional analysis of PP2C19 protein domains. (a) PP2C19 protein structure. The PP2C, cNMP-binding, and protein kinase domains are denoted by the black rectangles. The amino acid residues contained in each construct are indicated in parentheses. (b) Phototropic responses of the etiolated hypocotyls of wild-type (Col-0) seedlings, *pp2c19-1* seedlings, and transgenic *pp2c19-1* plants harboring the indicated variant of *35Spro::PP2C19*. Two-day-old etiolated seedlings grown along the surface of vertically oriented agar medium containing 1 µM NPA were irradiated with unilateral BL at 0.17 µmol m^–2^ s^–1^ for 6 h. Hypocotyl curvature of 36–46 seedlings was measured. The box plots’ components are described in the legend of Fig. 1a. Different letters (a–c) indicate statistically significant differences, and the same letters mark statistically nonsignificant differences (Tukey–Kramer multiple comparisons test, *P* < .05). (c) Phototropic responses of the etiolated hypocotyls of wild-type (Col-0) seedlings, *pp2c19-1* seedlings, and transgenic *pp2c19-1* plants harboring the indicated variant of *35Spro::PP2C19* and the *PP2C19pro::VENUS-PP2C19*. Two-day-old etiolated seedlings grown along the surface of vertically oriented agar medium containing 1 µM NPA were irradiated with unilateral BL at 0.17 µmol m^–2^ s^–1^ for 6 h. Hypocotyl curvature of 16–41 seedlings was measured. The box plots’ components are described in the legend of Fig. 1a. Different letters (a–c) indicate statistically significant differences, and the same letters mark statistically nonsignificant differences (Tukey–Kramer multiple comparisons test, *P* < .05).

The PP2C19 protein possesses the PP2C domain at the N-terminus, two cNMP-binding domains in the middle, and the protein kinase domain at the C-terminus. A previous study reported that the rice ortholog, cyclic guanosine monophosphate (cGMP)-dependent protein kinase (PKG; 65% amino acid sequence identity with PP2C19), has dual functions as a cGMP-dependent protein kinase and a cGMP-inhibited protein phosphatase and is involved in gibberellin-induced seed germination and salt stress response (Shen et al. 2019). In the present study, we investigated the physiological functions of each domain of the PP2C19 protein in hypocotyl phototropism. First, we generated *pp2c19* transgenic lines expressing the PP2C19 proteins with amino acid substitutions in each highly conserved motif of the PP2C domain, cNMP-binding domains, and the protein kinase domain (Fig. 2a, Supplementary Fig. S2). The PP2C19^D344N^ mutant carries the mutation of catalytic Asp (D) at residue 344 to Asn (N) in motif 8 of the PP2C domain (Tanoue et al. 2013). The PP2C^G565A^ and PP2C19^G707A^ mutants carry the mutation of the conserved Gly (G) at residues 565 and 707, respectively, to Ala (A) in motif 2 of the cNMP-binding domain (McCue et al. 2000). The PP2C19^D923N^ and PP2C19^T937A^ mutants carry the mutation of catalytic D923 to Asn and the mutation of phosphorylated Thr (T) at residue 937 to Ala in the activation loop of the protein kinase domain, respectively (Pereira et al. 2011). Not only the *pp2c19* transgenic lines expressing the PP2C19 wild-type proteins (PP2C19^WT^) but also PP2C19^G565A^, PP2C19^G707A^, PP2C19^D923N^, and PP2C19^T937A^ showed recovery of second positive phototropism on NPA-containing agar medium (Fig. 2b). In contrast, transgenic lines expressing PP2C19^D344N^ showed an abnormality similar to that in the *pp2c19-1* mutants, although the expression of PP2C19^D344N^ proteins was confirmed in these lines (Supplementary Fig. S3). Furthermore, not only the deletion mutants of the protein kinase domain (PP2C19^ΔKD^) but also the deletion mutants of the protein kinase domain and both cNMP-binding domains (PP2C19^ΔcNMP/KD^) showed complementation of the *pp2c19* phenotype in the phototropic response (Fig. 2a and c). These results indicated that the PP2C domain is necessary and sufficient for PP2C19 function in hypocotyl phototropism.

### Phenotypic analyses of the *pp2c19* mutants

**Figure 3.**
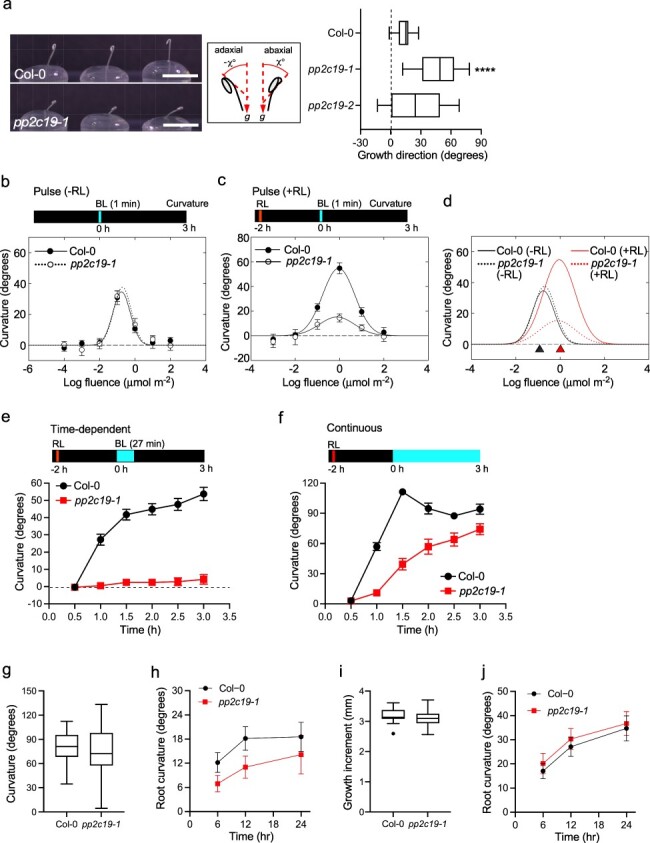
Phenotypic analysis of growth patterns in wild-type seedlings and *pp2c19* mutants. (a) Direction of hypocotyl growth in etiolated seedlings of wild-type (Col-0) seedlings and *pp2c19* mutants on agar in PCR tubes under darkness (right panel). The images on the left show typical seedlings of wild-type seedlings and *pp2c19-1* mutants on agar in PCR tubes. Scale bar = 5 mm. The central panel illustrates the degree of hypocotyl growth angle to the abaxial side (*χ*°) and the adaxial side (−*χ*°) of the hook. The box plots’ components are described in the legend of Fig. 1a. Asterisks indicate significant differences compared with the Col-0 (****P < .0001) as determined using one-way analysis of variance followed by Dunnett’s multiple comparison test. (b–f) Hypocotyl phototropism. Two-day-old etiolated seedlings grown on the agar inPCR tubes were mock preirradiated (b) or irradiated with overhead RL (RL: c–f) for 2 min at 20 µmol m^–2^ s^–1^ 2 h before BL irradiation. BL irradiation of the hypocotyl was administered perpendicularly to the plane of the hook to induce a phototropic curvature, as described previously (Haga and Sakai 2012). (b) Pulse-induced, first positive phototropism without RL pretreatment. Etiolated seedlings were stimulated with unilateral BL for 1 min at the indicated fluence. Hypocotyl curvature was determined at 3 h after the onset of BL irradiation. Data shown are mean ± standard error (SE) from eight seedlings. (c) Pulse-induced, first positive phototropism with RL pretreatment. Etiolated seedlings were stimulated with unilateral BL for 1 min at the indicated fluence. Hypocotyl curvature was determined at 3 h after the onset of BL irradiation. Data shown are mean ± SE from eight seedlings. (d) Summary of fluence-response curves (Fig. 3b and c) of pulse-induced phototropism in wild-type (Col-0) seedlings and *pp2c19-1* mutants. Red and black arrowheads show the peak of the fluence-response curves with or without RL pretreatment, respectively. (e) Time-course analysis of time-dependent phototropism. Following RL pretreatment, seedlings were stimulated with unilateral BL at 0.17 µmol m^–2^ s^–1^ for 27 min and then incubated under darkness. Hypocotyl curvature was determined at the indicated time points. Data shown are mean ± SE from 10 seedlings. (f) Time-course analysis of continuous light-induced phototropism. Following RL pretreatment, seedlings were stimulated with unilateral BL at 0.17 µmol m^–2^ s^–1^, and hypocotyl curvature was determined at the indicated time points. Data shown are mean ± SE from 10–12 seedlings. (g) Hypocotyl gravitropism. Following RL pretreatment, seedlings grown on the agar in PCR tubes were displaced horizontally under darkness. Gravistimulation was administered perpendicularly to the plane of the hook, and hypocotyl curvature of 23–24 seedlings was determined at 24 h after the onset of gravistimulation. The box plots’ components are described in the legend of Fig. 1a. (h) Time-course analysis of root phototropism. Two-day-old etiolated seedlings grown along the surface of vertically oriented agar medium were stimulated with unilateral BL at 100 µmol m^–2^ s^–1^, and root curvature was determined at the indicated time points. Data shown are means ± SE from 22 seedlings. (i) Elongation of hypocotyls. Two-day-old etiolated seedlings grown on the agar in PCR tubes were incubated under darkness for 8 h, and the growth increment of hypocotyls was determined by measuring the hypocotyl lengths of 22 seedlings. The box plots’ components are described in the legend of Fig. 1a. (j) Time-course analysis of root gravitropism. Two-day-old etiolated seedlings grown along the surface of vertically oriented agar medium were rotated at 90° under darkness, and root curvature was determined at the indicated time points. Data shown are mean ± SE from 14 seedlings. The differences between wild-type (Col-0) seedlings and *pp2c19-1* mutants in Fig. 3g–j were not statistically significant (two-tailed Mann–Whitney *U* test, *P* < .05).

The phototropic responses of the *pp2c19* mutants were further analyzed using PCR tubes, as described in previous studies (Haga and Sakai 2012, Haga and Kimura 2019). Many 2-day-old etiolated seedlings of the *pp2c19* mutants showed a hypocotyl bending toward the abaxial (rear) side of the hook in the absence of light irradiation (Fig. 3a). Therefore, seedlings showing upward growth were selected before phototropic or gravitropic stimulation, and these stimulation treatments were administered perpendicular to the plane of the hook to avoid an effect of hypocotyl bending in the abaxial–adaxial axis of the hook. When pulse-induced, first positive phototropism was observed without RL pretreatment, the fluence-response curve of *pp2c19-1* mutants was almost identical to that of the wild type (Fig. 3b). Conversely, when first positive phototropism was observed in the presence of RL pretreatment, the enhancement of hypocotyl curvatures with RL pretreatment was not observed in the *pp2c19* mutant, in contrast to the wild type (Fig. 3c and d). A shift in the fluence with the peak of the fluence-response curve from 0.1 to 1 μmol m^−2^ with RL pretreatment was observed in wild-type seedlings and the *pp2c19* mutants (Fig. 3d).

Next, time-dependent phototropism and continuous light-induced phototropism were examined in the presence of RL pretreatment. The *pp2c19* mutants showed a defect in time-dependent phototropism under the light condition examined (Fig. 3e). Regarding continuous light-induced phototropism, slower hypocotyl bending was observed in the *pp2c19* mutants, with a curvature similar to that of the wild type after 3 h of BL irradiation (Fig. 3f), as was the case with the vertically oriented agar medium (Fig. 1d). No statistically significant differences were detected in hypocotyl gravitropism and root phototropism of the wild type and *pp2c19-1* mutant; however, the gravitropic curvature of *pp2c19-1* mutant hypocotyls showed greater variation, and the phototropic curvature of *pp2c19-1* mutant roots was slightly smaller than that of wild-type roots (Fig. 3g and h). Furthermore, we could not detect any abnormalities in the growth of *pp2c19-1* hypocotyls and root gravitropism (Fig. 3i and j). Taken together, the results of the phenotypic analyses suggest that PP2C19 has a function not only in second positive phototropism (Fig. 3e and f) but also in the regulation of the direction of growth of etiolated hypocotyls in the dark (Fig. 3a) and in the promotion of hypocotyl curvature with RL pretreatment in first positive phototropism (Fig. 3c).

### Immunoblot analysis of *NPH3 in the pp2c19* mutants

**Figure 4.**
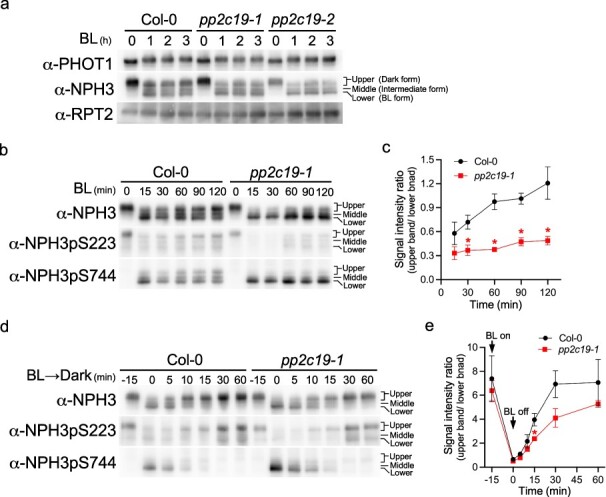
Immunoblot analyses of PHOT1, NPH3, and RPT2 proteins. (a) Time-course analysis of changes in PHOT1, NPH3, and RPT2 proteins. Two-day-old etiolated seedlings of wild-type (Col-0) seedlings and *pp2c19-1* and *pp2c19-2* mutants were preirradiated with overhead RL irradiation for 2 min at 20 µmol m^–2^ s^–1^ 2 h before BL irradiation and subsequently irradiated with unilateral BL at 0.17 µmol m^–2^ s^–1^ for the indicated periods. Proteins (10 μg) extracted from the seedlings were separated via 7.5% SDS-PAGE, followed by immunoblotting with the indicated antibodies. (b) Time-course analysis of NPH3 protein changes in response to BL irradiation. Two-day-old etiolated seedlings of wild-type (Col-0) seedlings and *pp2c19-1* mutants were preirradiated with overhead RL irradiation for 2 min at 20 µmol m^–2^ s^–1^ 2 h before BL irradiation and subsequently irradiated with unilateral BL at 0.17 µmol m^–2^ s^–1^ for the indicated periods. (c) Signal intensity ratio of the upper band to the lower band in the immunoblotting using the anti-NPH3 antibody in Fig. 4b. Data shown are mean ± SE from three independent experiments. Asterisks indicate statistically significant differences (two-tailed Student’s *t* test, *P* < .05). Signal intensity ratios at 0 min were significantly larger than those at other time points; therefore, they were omitted from the graph. (d) Time-course analysis of NPH3 protein changes in response to BL irradiation and subsequent dark incubation. Two-day-old etiolated seedlings of wild-type (Col-0) seedlings and *pp2c19-1* mutants (−15 min) were irradiated with unilateral BL at 0.17 µmol m^–2^ s^–1^ for 15 min (0 min) and then incubated under darkness for the indicated periods. (e) Signal intensity ratio of the upper band to the lower band in the immunoblotting using the anti-NPH3 antibody in Fig. 4d. Data shown are mean ± SE from three independent experiments. Asterisk indicates a statistically significant difference (two-tailed Student’s *t* test, *P* < .05).

The involvement of PP2C19 in the phosphorylation modification of PHOT1 and NPH3 was analyzed by immunoblotting. Similar to the protocol used for the induction of the phototropic response (Fig. 3f), 2-day-old etiolated seedlings were preirradiated with RL at 2 h before unilateral BL irradiation at 0.17 μmol m^−2^ s^−1^. The expression patterns of PHOT1, NPH3, and RPT2 were then examined (Fig. 4a). Owing to their autophosphorylation (Christie et al. 1998, Haga et al. 2015), PHOT1 proteins exhibited slower electrophoretic mobility in wild-type seedlings and *pp2c19* mutants exposed to BL irradiation for 1–3 h. In wild-type seedlings and *pp2c19* mutants exposed to BL irradiation for 1–3 h, NPH3 proteins appeared as three distinct bands, reflecting different levels of electrophoretic mobility due to dephosphorylation. Herein, we defined the upper, lower, and middle bands as the dark, BL, and intermediate forms of NPH3, respectively (Fig. 4a). The shift from the NPH3 dark form to the NPH3 BL form in response to BL irradiation differed between wild-type seedlings and *pp2c19* mutants. Most NPH3 proteins extracted from wild-type seedlings and *pp2c19* mutants under BL conditions were located on the upper and lower sides of the mobility, respectively. RPT2 expression was maintained at a constant level when etiolated seedlings were preirradiated with RL (Fig. 4a), similar to the results of a previous study (Haga et al. 2015); no specific changes in the expression level of RPT2 were observed between the wild-type seedlings and *pp2c19* mutants (Fig. 4a).

We further analyzed the phosphorylation modification of NPH3. Immunoblotting with anti-NPH3 antibody against the proteins of wild-type etiolated seedlings showed a transition from the dark form to the BL form of NPH3 after 15 min of BL irradiation, although some dark and intermediate forms were also detected (Fig. 4b). Prolong BL irradiation decreased the BL form and recovered the dark form (Fig. 4b and c). In contrast, the *pp2c19* mutant exhibited a more pronounced shift to the BL form than wild-type seedlings after 15 min of BL irradiation, with no recovery to the dark form observed over time (Fig. 4b and c). Moreover, antibodies recognizing the phosphorylated S223 (pS223) and S744 (pS744) were used to assess the phosphorylation status of NPH3 IDRs and S744, respectively. In wild-type seedlings, pS223 was identified as the upper band in electrophoretic mobility, whereas pS744 was undetectable under dark conditions (Fig. 4b); this observation was consistent with previous studies (Tsuchida-Mayama et al. 2008, Sullivan et al. 2021). After 15 min of BL irradiation, pS223 was nearly absent but became partially detectable after ≥30 min of BL irradiation. pS744 was detected as the lower band of NPH3 proteins in electrophoretic mobility after 15 min of BL irradiation and gradually increased in the upper and middle bands as irradiation continued. In *pp2c19* mutants, pS223 was detectable in darkness but not under BL conditions (Fig. 4b), whereas pS744 was strongly present in the lower band but not in the middle or upper bands under BL conditions.

Furthermore, NPH3 phosphorylation recovery was observed when seedlings were returned to darkness following 15 min of BL irradiation at 0.17 μmol m^−2^ s^−1^, without RL pretreatment to avoid inhibition of NPH3 BL form formation (Haga et al. 2015). In wild-type seedlings, recovery of the NPH3pS223 dark form from the NPH3pS744 BL form began at 5 min of dark incubation, with the NPH3pS744 BL form disappearing by 30 min (Fig. 4d). In *pp2c19* mutants, NPH3pS223 dark form recovery began at 10 min of dark incubation, with NPH3pS744 disappearing by 30 min (Fig. 4d). A statistically significant difference in the ratio of upper and lower band signal intensities between the wild-type seedlings and *pp2c19* mutants at 15 min was observed via immunoblotting with the anti-NPH3 antibody (Fig. 4e), indicating that the recovery of the NPH3pS223 dark form in *pp2c19* mutants was delayed compared to the wild type. Taken together, these results indicate that PP2C19 functions in the reverse reaction converting the NPH3 BL form to the NPH3 dark form, which may explain why *pp2c19* mutants showed abnormalities in second positive phototropism, in particular a defect of time-dependent phototropism (Fig. 3e). Thus, a partial recovery of the NPH3 dark form from the NPH3 BL form during 27 min of BL irradiation appeared to be insufficient for time-dependent phototropism in *pp2c19*. On the other hand, the recovery of the NPH3 dark form from the NPH3 BL form under dark conditions occurred slightly later in the *pp2c19* mutant than in the wild type (Fig. 4d and e), suggesting that the reverse reaction occurs even in the absence of PP2C19 and that an equilibrium state more biased toward the BL form is produced in the *pp2c19* mutants compared with the wild type. A bias in the equilibrium reaction of the NPH3 dark form and the NPH3 BL form conversion may retard the differential growth in continuous light-induced, second positive phototropism in the *pp2c19* mutants (Fig. 3f).

### Subcellular localization of PP2C19

**Figure 5.**
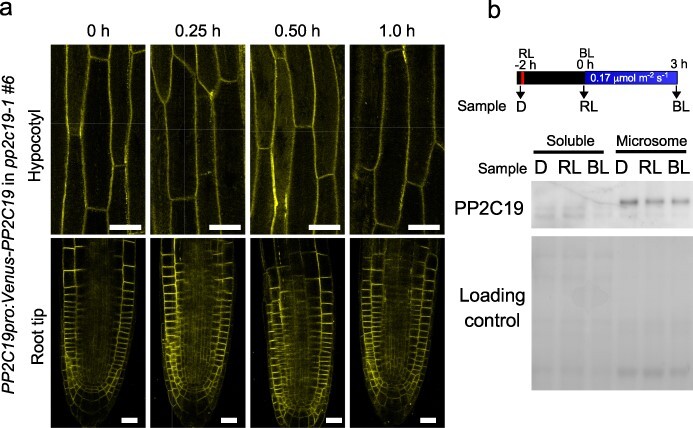
Localization of PP2C19 proteins and effects of light treatments. (a) Confocal fluorescence microscopy images of transgenic *pp2c19-1* plants harboring the *PP2C19pro::VENUS-PP2C19*. Two-day-old etiolated seedlings were stimulated with BL at 0.17 µmol m^–2^ s^–1^, and VENUS fluorescence signals were observed with a microscope at the indicated times after the onset of BL irradiation. Scale bars = 20 μm. (b) Immunoblot analysis of PP2C19 proteins in the wild-type (Col-0) seedlings before and after phototropic stimulation. Two-day-old etiolated seedlings were preirradiated with overhead RL irradiation for 2 min at 20 μmol m^−2^ s^−1^ 2 h before BL irradiation and then irradiated with unilateral BL at 0.17 µmol m^–2^ s^–1^ for 3 h. Seedlings were collected before the RL pretreatment (sample D), before the BL irradiation (sample RL), and after the BL irradiation for 3 h (sample BL), as shown in an upper panel. Soluble proteins (soluble) and crude microsomal proteins (microsome) were prepared from collected seedlings, and proteins (2 μg) in each fraction were separated on 6% SDS-PAGE gels, followed by immunoblotting with anti-PP2C19 antibody. The PVDF membranes were stained using a Pierce reversible protein staining kit as a loading control.

Subcellular localization of PP2C19 was determined by fluorescence imaging of the yellow fluorescent protein VENUS-fused PP2C19. Transgenic *pp2c19-1* seedlings carrying the *PP2C19pro::VENUS-PP2C19* gene showed a phototropic response similar to that of wild-type seedlings on the surface of NPA-containing vertically oriented agar medium (Fig. 2c), indicating that this construct is functional. Clear fluorescence images of epidermal cells of the hypocotyl and root tips were obtained using 2-day-old etiolated seedlings (Fig. 5a). These images suggested that the VENUS-fused PP2C19 proteins were mainly localized to the plasma membrane, similar to phot1 and NPH3 (Sakamoto and Briggs 2002, Haga et al. 2015). The subcellular localization of PP2C19 on the plasma membrane was confirmed by cell fractionation. Immunoblot analysis of soluble and microsomal fractions after ultracentrifugation using anti-PP2C19 antibody showed that PP2C19 was present in the microsomal fraction before and after phototropic stimulation (Fig. 5b). Moreover, the expression levels of PP2C19 did not change after RL pretreatment and subsequent BL irradiation (Fig. 5b). These results indicate that PP2C19, similar to phot1 and NPH3, is localized to the plasma membrane and that its localization and expression level are not regulated by light, suggesting that PP2C19 directly or indirectly controls the phosphorylation of NPH3 on the plasma membrane.

## Discussion

In this study, we have attempted to elucidate the molecular mechanism of the continuous light-induced hypocotyl phototropism observed in the *pin3 pin7* mutants and identified a novel regulator of phototropic responses, PP2C19, which plays an important role in regulating NPH3 phosphorylation. In our previous studies, we found that the saturation of the BL-induced dephosphorylation reaction of phosphorylated Ser residues—including S223—in the IDRs of NPH3 coincides with the refractory state of the fluence-response curve and that the recovery of their phosphorylation status is required for second positive phototropism (Haga et al. 2015, Kimura et al. 2021). Sullivan et al. (2021) demonstrated that S744 of NPH3 is phosphorylated by BL-activated phot1, which promotes the phototropic responses. In the present study, we analyzed the phosphorylation modification patterns of NPH3 proteins using anti-NPH3, anti-pS223, and anti-pS744 antibodies. Few NPH3 proteins containing both pS223 and pS744 were detected, suggesting that the protein exists distinctly as the NPH3pS223 dark form with slow electrophoretic mobility in sodium dodecyl sulfate–polyacrylamide gel electrophoresis (SDS-PAGE) and as the NPH3pS744 BL form with fast electrophoretic mobility in SDS-PAGE. The NPH3 dark form present in the dark transiently disappears upon BL irradiation and shows partial recovery with time, whereas the NPH3 BL form appears upon BL irradiation and disappears upon return to the dark. In other words, the results showed the reversibility of the forward reaction—in which BL irradiation causes a change from the dark form to the BL form—and the reverse reaction—in which the BL form reverts to the dark form in the dark. Our molecular genetic analyses showed that PP2C19 promotes the reverse reaction. PP2C19 showed subcellular localization at the plasma membrane, similar to phot1 and NPH3. Future biochemical analysis of PP2C19 is needed to determine whether PP2C19 acts directly in the dephosphorylation of NPH3pS744 and the phosphorylation of S223 or indirectly in the regulation of their phosphorylation modifications.

**Figure 6.**
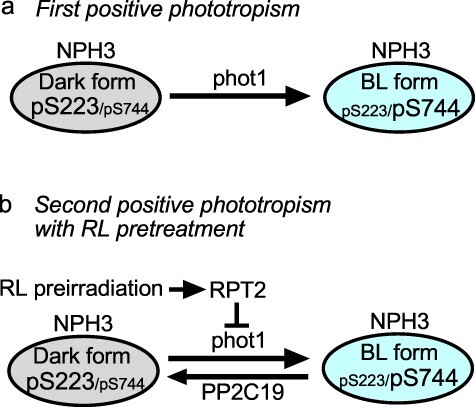
Hypothetical model for controlling NPH3 phosphorylation status. (a) Regarding the first positive phototropism, pulse BL-activated phot1 causes the forward reaction from the NPH3 dark form (in which pS223 is predominant and pS744 is minor) to the NPH3 BL form (in which pS223 is minor and pS744 is predominant), reaching saturation on the irradiated and shaded sides of the hypocotyl, resulting in the refractory state in the fluence-response curve. (b) Regarding the second positive phototropism, PP2C19 promotes the reverse reaction from the NPH3 BL form to the NPH3 dark form, whereas phot1 promotes the forward reaction, leading to an equilibrium state between the NPH3 dark and BL forms under continuous BL conditions. RL preirradiation suppresses the phot1 autophosphorylation activity through the *RPT2* induction, enhancing the reverse reaction from the NPH3 BL form to the NPH3 dark form. In *pp2c19* and *rpt2* mutants, the equilibrium shifts toward the NPH3 BL form, leading to their abnormalities in the second positive phototropism.

The abnormalities in second positive phototropism in *pp2c19* mutants can be attributed to the attenuation of the reverse reaction from the NPH3 BL form to the NPH3 dark form. Phototropic responses are thought to be caused by an activity gradient of phototropin signaling between the BL-irradiated and shaded sides of hypocotyls, and we hypothesize that the combination of gradients of phot1 kinase activity and NPH3 phosphorylation status influence the formation of a gradient of phototropin signaling activity, as in the photoproduct gradient hypothesis of the phot1–NPH3 complex (Haga and Sakai 2023). In etiolated seedlings, the NPH3 BL form is almost nonexistent; therefore, unilateral pulse BL irradiation activates the forward reaction from the NPH3 dark form to the NPH3 BL form through phot1 activity and generates gradients of phot1 activity and NPH3 phosphorylation (Fig. 6a), leading to first positive phototropism. When phot1 activity becomes high with BL irradiation at high fluences, the forward reaction is enhanced. This leads to depletion of the NPH3 dark form and decreases the NPH3 phosphorylation gradient, resulting in the refractory state of the fluence-response curve. Prolonged BL irradiation for 27–30 min leads to the time-dependent, second positive phototropism with a slight recovery of the NPH3 dark form (Figs 3e and 4b and c), suggesting that prolonged BL irradiation leads to an equilibrium state of the reversible reactions of NPH3 phosphorylation modification (Fig. 6b), resulting in second positive phototropism. In this context, the phenotypes of *rpt2* and *pp2c19* are easy to understand: the *rpt2* mutation enhances the phot1 kinase activity and the forward reaction (Haga et al. 2015, Kimura et al. 2020), and the *pp2c19* mutation attenuates the reverse reaction (Fig. 4), both shifting the equilibrium state toward NPH3 BL form formation. This is thought to result in abnormalities in the phot1 signaling gradient and second positive phototropism in *rpt2* and *pp2c19* mutant hypocotyls. The *pin3 pin7* double mutations enhanced the abnormality of second positive phototropism in the *pp2c19* mutants, suggesting that PIN3 and PIN7 function not only in first positive phototropism but also in second positive phototropism. The need for a gradient of phot1 signaling may be higher for hypocotyl phototropism dependent on the PIN3/PIN7-independent pathway. Alternatively, the need for the asymmetric auxin distribution generated by PIN3 and PIN7 may be higher for hypocotyl phototropism in *pp2c19* mutants.

The *pp2c19* mutants showed two other abnormalities in addition to those in second positive phototropism. First, although first positive phototropism itself occurred normally, the hypocotyl curvature-promoting effect of RL pretreatment was not shown in the mutants. Previous research suggests that the hypocotyl curvature-promoting effect of RL pretreatment is mediated by up-regulation of PIN proteins and down-regulation of PINOID AGC kinases (Haga et al. 2014). Second, *pp2c19* hypocotyls exhibited a phenotype of hypocotyl bending toward the abaxial (rear) side of the hook in the absence of light irradiation. This phenotype is similar to that of ABC subfamily B auxin transporter ABCB19 loss-of-function seedlings irradiated with RL (Sakai et al. 2014); however, in the case of the *abcb19* mutant, second positive phototropism was rather accelerated (Noh et al. 2003). These phenotypes are not explained by abnormalities in NPH3 phosphorylation modification; the *pp2c19* mutant showed no abnormalities in gravitropism of hypocotyls or roots, suggesting that the auxin-dependent induction of differential growth is not itself abnormal in the *pp2c19* mutants, at least in the dark. PP2C19 has recently been reported as one of the potential hubs in the network of auxin-triggered phospho-responses (Kuhn et al. 2024). Furthermore, the rice ortholog PKG has been demonstrated to have pleiotropic effects on plant growth and environmental responses, including gibberellin-induced seed germination and the salt stress response (Shen et al. 2019). It will be interesting to examine whether phot1 controls PP2C19 function and whether PP2C19 has other functions in the environmental responses of *Arabidopsis*.

## Materials and Methods

### Plant materials and growth conditions

*Arabidopsis thaliana* seeds of wild type [Columbia (Col)-0], *pin3-5 pin7* (Salk_048791), and *d6pk* (Salk_061847) mutants were as described previously (Haga and Sakai 2012, Haga et al. 2018). Seeds of *pin3* [ET5573: a Ds transposon insertion line generated in the Landsberg *erecta* (L*er*) background], *pin7-3* (L*er* background), *pp2c19-1* (SALK_108016), *pp2c19-2* (SALK_064474), *pp2c19-3* (SALK_138956), and *pp2c19-4* (GK918D02) were obtained from the ABRC (Sundaresan et al. 1995, Alonso et al. 2003, Kleinboelting et al. 2012). The *pin3* (ET5573) *pin7-3* double mutant was generated by crossing.

Transgenic plants were generated as follows: the 3′ region of *PP2C19* cDNA was generated by RT-PCR using the PCR primers PP2C19FW12 and PP2C19RV5 and poly(A) RNA of *Arabidopsis* seedlings, and the 5′ region of *PP2C19* genomic DNA was generated by PCR using the PCR primers PP2C19FW5 and PP2C19RV10 and genomic DNA of *Arabidopsis* seedlings. Then, both PCR products were mixed and amplified by PCR using the primers PP2C19FW5 and PP2C19RV5 to generate the *PP2C19* cDNA with the first intron (Supplementary Table S1), which was cloned into the pENTR/D-TOPO plasmid vector (Thermo Fisher Scientific). This construct was used as the *PP2C19* wild-type construct (*PP2C19^WT^*). Mutant genes of *PP2C19^D344N^, PP2C19^G565A^, PP2C19^G707A^*, PP2C19^D923N^, and *PP2C19^T937A^* were generated from *PP2C19^WT^* by PCR-based site-directed mutagenesis using PCR primers, as shown in Supplementary Table S1. The 3′ deletion mutants of *PP2C19^ΔKD^* and *PP2C19^ΔcNMP/KD^* were also generated from *PP2C19^WT^* by PCR using the PP2C19FW5 and PP2C19del1-783RV primers and the PP2C19FW5 and PP2C19del1-475RV primers, respectively (Supplementary Table S1). All PCR-amplified genes were cloned into the pENTR/D-TOPO vector. pENTR/D-TOPO constructs were recombined with the pH35GS binary vector (Kubo et al. 2005) using the Gateway LR reaction (Thermo Fisher Scientific) to obtain *PP2C19* genes fused with the cauliflower mosaic virus 35S promoter (various *35Spro::PP2C19* constructs shown in Fig. 2). The constructs were transformed into the *pp2c19-1* mutant via the floral dip method using *Agrobacterium tumefaciens*-mediated transformation, following a previously described protocol (Clough and Bent 1998).

The cloning of *PP2C19pro::VENUS-PP2C19* was performed as follows: the 5′ region of the *PP2C19* genomic DNA was amplified using the PCR primers PP2C19B1 and PP2C19B4 (from −2208 to +12 bp from the start codon; Supplementary Table S1) and cloned into pDONR221P1-P4 (Thermo Fisher Scientific). The *PP2C19^WT^* gene was amplified using the PCR primers PP2C19attB3 and PP2C19attB2 (Supplementary Table S1) and cloned into pDONR221P3-P2 (Thermo Fisher Scientific). The VENUS cDNA cloned into pDONR221P4r-P3r was used, as described in a previous study (Haga et al. 2014). Three different pDONR constructs were recombined with the pGWB501 binary vector (Nakagawa et al. 2007) using the Multi-Gateway LR reaction (Thermo Fisher Scientific) to obtain the *PP2C19pro::VENUS-PP2C19* construct. The construct was transformed into the *pp2c19-1* mutants via *A. tumefaciens*-mediated transformation (Clough and Bent 1998).

In most analyses, the etiolated seedlings were grown along the surface of vertically oriented agar medium (Haga and Kimura 2019). The seeds were sown on a square plastic Petri dish with half-strength Okada and Shimura medium containing 1.5% (w/v) agar and kept at 4°C for 3–5 days. If necessary, NPA dissolved in dimethyl sulfoxide was added to the medium, and seed direction was adjusted with the hook of the embryo directed upward on the vertically oriented agar medium with NPA, as described previously (Nagashima et al. 2008). After induction of germination, the prepared seeds were incubated at 21–23°C for 2 days in complete darkness. For physiological experiments (Fig. 4a–h), the seeds were sown in 0.2 ml plastic tubes filled with 1.5% agar medium, placed in a black plastic box, and kept at 4°C for 3–5 days, as described previously (Haga and Kimura 2019). After induction of germination, the prepared seeds were incubated at 21–23°C for 2 days in complete darkness. Seedlings were selected on the basis of length (3–5 mm) and the direction of growth (straight upward) of the hypocotyls. During the experiments, seedlings were kept in a black plastic box under high humidity until used. Then, the etiolated seedlings were used for analyses. Experimental procedures were performed under dim green light.

### Map-based cloning

*DP3* [EMS-treated *pin3-5 pin7* (Salk_048791): Col-0 background] was crossed with the *pin3* (ET5573) *pin7-3* double mutant (L*er* background), and F2 seedlings were selected by screening for a defect in second positive phototropism. Genomic DNA was isolated from individual F2 seedlings and amplified using primers specific for various PCR-based SSLP markers (Supplementary Table S1), which were designed from SNPs stored in the Cereon Arabidopsis Polymorphism Collection (Jander et al. 2002). The seedlings were then assayed for Col- and L*er*-specific polymorphisms. SMART software was used to analyze the structure of the PP2C19 protein (http://coot.embl-heidelberg.de/SMART: Schultz et al. 1998).

### Whole-genome sequencing

Genomic DNA was isolated from seedlings of *DP1* and *DP3* mutants using the DNeasy plant mini kit (Qiagen). Library preparation was performed according to the manufacturer’s instructions (Nextera; Illumina), and sequencing was performed on an Illumina NextSeq 550. Reads were aligned to the *Arabidopsis* Col-0 reference genome (TAIR 10) using CLC Genomic Workbench software (Qiagen).

### Immunoblot analysis

Following the light treatments, whole seedlings were harvested and immediately dipped in liquid nitrogen. The frozen samples were finely pulverized using a mixer mill (TissueLyser II; Qiagen), and total crude proteins were extracted with 1 × SDS gel loading buffer. The extracts were boiled and then centrifuged at 10 000 × g at 4°C for 10 min to remove cell debris. The samples were separated on SDS-PAGE gels for the subsequent immunoblot analysis. Fractions of soluble proteins and crude microsomal proteins were prepared using a previously reported protocol (Haga et al. 2015). A polyclonal rabbit antibody against PP2C19 was raised by using a His-tagged C-terminal fragment of PP2C19 containing amino acids 471–1094 (PP2C19Cter) as an antigen. The 3′ region of *PP2C19^WT^* was amplified using the PCR primers PP2C19FW14 and PP2C19RV5 and cloned into the pENTR/D-TOPO vector (Thermo Fisher Scientific). The pENTR/D-TOPO construct was recombined with the pDEST17 vector (Thermo Fisher Scientific) using the Gateway LR reaction. The pDEST17-*PP2C19Cter* construct was transformed into the *Escherichia coli* BL21-Al strain (Thermo Fisher Scientific), and His-tagged PP2C19Cter proteins were expressed according to the manufacturer’s instructions (Thermo Fisher Scientific). Anti-NPH3pS744 antibody was prepared as described previously (Sullivan et al. 2021). Anti-PHOT1, anti-RPT2, anti-NPH3, anti-NPH3pS223, and horseradish peroxidase-conjugated anti-rabbit IgG antibodies were used as described in previous studies (Tsuchida-Mayama et al. 2008, Haga et al. 2015). Horseradish peroxidase activity was detected using the SuperSignal West Femto Maximum Sensitivity Substrate kit (Thermo Fisher Scientific) and imaged with an Image Quant LAS4000 Mini instrument (GE Healthcare). As a loading control, the polyvinylidene fluoride membranes were stained using the Pierce reversible protein staining kit (Thermo Fisher Scientific). The results were confirmed using independent samples. Signal intensities of bands were quantified using ImageJ (Schneider et al. 2012, Ohgane and Yoshioka 2019).

### Induction of phototropism and gravitropism

Hypocotyl phototropism was analyzed as described in a previous study (Haga and Kimura 2019). Phototropic stimulation (470 ± 30 nm, LED-B; Eyela) was applied through two layers of blue filter (no. 72 film; Tokyo Butai Shomei). The fluence rate was controlled with neutral-density plastic filters (Fujifilm). For RL pretreatment, the seedlings were irradiated with overhead RL (660 ± 20 nm, LED-R; Eyela) at 20 μmol m^−2^ s^−1^ for 2 min. For the analysis of hypocotyl phototropism using square Petri dishes, hypocotyls of 2-day-old etiolated seedlings were oriented with forceps so that they were aligned vertical to gravity and then irradiated with unilateral BL for 6 h. Images of seedlings were taken after the onset of the stimulation. For the analyses of hypocotyl phototropism and gravitropism using PCR tubes, selected seedlings were irradiated with unilateral BL under various conditions and were gravistimulated by a 90° rotation, respectively. The direction of the tropic stimulation was perpendicular to the plane of the hook (Haga and Kimura 2019). To induce root phototropism, seedlings prepared in Petri dishes were stimulated with unilateral BL at 100 μmol m^−2^ s^−1^ for 24 h. For induction of hypocotyl and root gravitropism, etiolated seedlings were displaced by 90° from the vertical orientation under darkness for 24 h.

### Measurement of curvature and growth

Images of dark-grown seedlings were recorded with a digital camera (D5000; Nikon or EOS RP; Canon), from which the UV/infrared light cut filter was removed (IDAS Division, ICAS Enterprises) under infrared illumination (IRDR-110; Nissen Electronics). The angle and length of the hypocotyls and roots were measured with an e-Ruler (Haga and Sakai 2012) or Canvas 11 software (ACD Systems International Inc.).

### Confocal laser scanning microscopy

VENUS signals were detected with a TCS-SP5 confocal laser scanning microscope (Leica Microsystems). The seedlings were excited with an argon laser at 514 nm, and the fluorescence signals were detected using a spectral detector set at 525–560 nm. The same settings (laser power and HyD photon counting mode) were used for a direct comparison of fluorescence intensity. Scans were performed at a 2048 × 2048 pixel resolution with repeated scanning of two lines.

## Supplementary Material

pcae141_Supp

## Data Availability

The data underlying this article will be shared on reasonable request to the corresponding author.
