# Supplementary material for: Protein phosphatase PP2C19 controls hypocotyl phototropism through the phosphorylation modification of NONPHOTOTROPIC HYPOCOTYL3 in Arabidopsis
Source: Plant Cell Physiol. 2024 Nov 28;66(1):23–35. doi: 10.1093/pcp/pcae141 (PMC11775391; doi:10.1093/pcp/pcae141)
Supplement: pcae141_Supp [file pcae141_supp.zip › suppl_data/pcp-2024-e-00216-File008.pdf]

Table S1. List of primers used in this study

No.	Primer	Sequence (5′→3′)
1	PP2C19FW12	GATCCTCGAGATGCTTGTGC
2	PP2C19RV5	GTCTTACCATTCTTCAAGCCAG
3	PP2C19FW5	CACCATGGGTTGTGCTTATTCCAAAAC
4	PP2C19RV10	GCACAAGCATCTCGAGGATC
5	PP2C19D344NFW	GTTGCTAGTAATGGCGTATTTGAGTTCATCTC
6	PP2C19D344NRV	CGCCATTACTAGCAACCACAAAGAAAGG
7	PP2C19G565AFW	CATCATTCGGTGAACTCGCCTTAATGC
8	PP2C19G565ARV	CGAGTTCACCGAATGATGATTGTTTCTCAGC
9	PP2C19G707AFW	GTTACTTTGCTGAATGGGCTCTTCTTGG
10	PP2C19G707ARV	GCCCATTCAGCAAAGTAACTTCCTTCATGC
11	PP2C19D923NFW	ACAGATTGTAAACTTCAGATTCGCCAAGAAATTG
12	PP2C19D923NRV	TCTGAAGTTTACAATCTGTAGATATCCGGATTG
13	PP2C19T937AFW	CGAACATTTGCAATCTGTGGAAATGCAG
14	PP2C19T937ARV	CACAGATTGCAAATGTTCGTTCCCCGG
15	PP2C19del1-783RV	GTTCATTCCAGGTCTGCAAGAGTG
16	PP2C19del1-475RV	TCTCAGGTTTTTCTGTGGGCTGG
17	PP2C19attB1	GGGGACAAGTTTGTACAAAAAAGCAGGCTTCGCCACATCTTTTCTCAC
18	PP2C19attB4	GGGGACAACTTTGTATAGAAAAGTTGGGTGAGCACAACCCATCACTGAG
19	PP2C19attB3	GGGGACAACTTTGTATAATAAAGTTGCTTATTCCAAAACTTGTATTGGTC

20	PP2C19attB2	GGGGACCACTTTGTACAAGAAAGCTGGGTATTACCATTCTTCAAGCCAGTTTTG
21	F6P27 SSLP Fw	TTGGCAGAATGGTTGAGTTC
22	F6P27 SSLP Rv	ACTGCGTCAACCTTCCGTAG
23	F27A10 SSLP Fw	GATAGGTAACCTTTCTCATACAGC
24	F27A10 SSLP Rv	GTTTGTGTACGGGATGAATCACG
25	F7D8 SSLP Fw	GCTCACTCCAAATCGAGAAG
26	F7D8 SSLP Rv	GTAGACTTTCTGTTTTGAACTTCC
27	F3P11 SSLP Fw	CTATGACCAGTTGTACCAATGTGG
28	F3P11 SSLP Rv	ATTCTGAAAGCTTACTGAGCTAAG
29	PP2C19FW14	CACCGCCCACAGAAAAACCTGGG

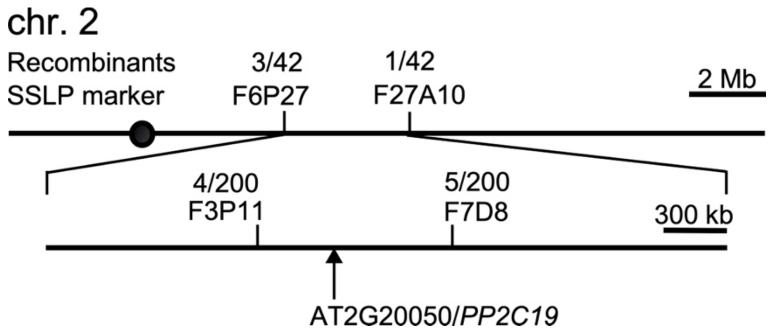

Fig. S1 Map position of the *PP2C19* gene. Numerals above SSLP markers indicate the number of recombinant chromosomes found in 42 or 200 chromosomes of F2 progeny exhibiting the *DP3* mutant genotype.

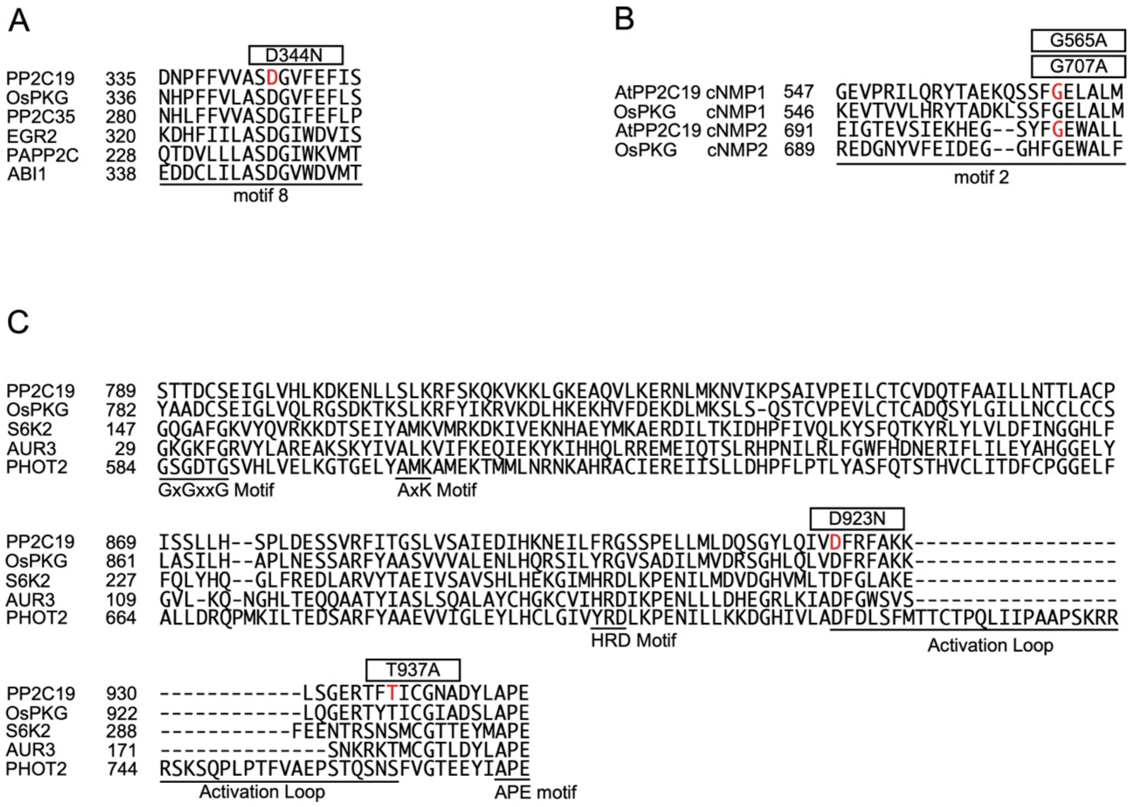

Fig. S2 Alignments of conserved regions of PP2C, cNMP-binding, and protein kinase domains. Red letters indicate amino acid substitution mutations in Fig. 2. (A) PP2C domain alignment. The motif 8 of PP2C domains of PP2C19, OsPKG (ortholog of PP2C19 in rice), PP2C35, EGR2 (AT5G27930), PAPP2C (AT1G22280) and ABI1 (AT4G26080) are aligned. (B) cNMP-binding domain alignment. The motif 2 of cNMP-binding domains of PP2C19 and OsPKG are aligned. (C) Protein kinase domain alignment. The kinase domains of PP2C19, OsPKG, S6K2 (AT3G08720), AUR3 (AT2G45490), PHOT2 (AT5G58140) are aligned from their GxGxxG motifs through the activation loop, to the APE motifs.

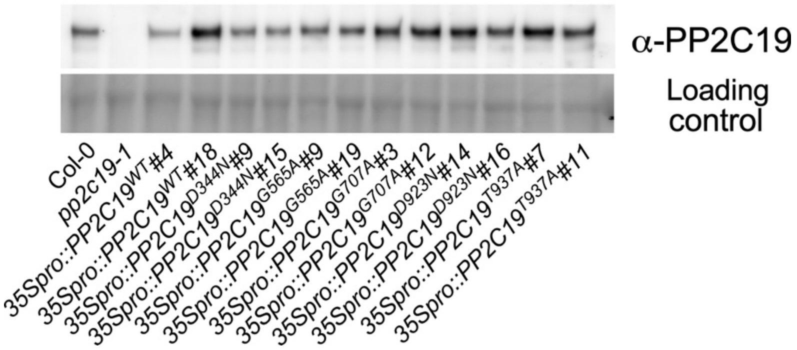

Fig. S3 Expression levels of PP2C19 proteins in the transgenic *pp2c19-1* plants harboring the *35Spro::PP2C19*. Immunoblotting for the PP2C19 protein. Total protein extracts of 2-day-old etiolated seedlings of Col-0, *pp2c19-1* and transgenic *pp2c19-1* plants harboring the indicated variant of *35Spro::PP2C19* were analyzed: 20 μg of each protein preparation was separated by 6% SDS–PAGE and immunoblotted using anti-PP2C19 antibodies**.**
